# Intrabiliary injection of indocyanine green dye (ICG) versus intravenous injection for patients undergoing laparoscopic cholecystectomy for symptomatic gallbladder diseases: A systematic review and meta-analysis

**DOI:** 10.1186/s12893-026-03529-4

**Published:** 2026-02-16

**Authors:** Mohamed Gamal, Sohieb Hedawy

**Affiliations:** 1 Faculty of Medicine, Assiut University, Assiut, Egypt; 2 Faculty of Medicine, Al-Azhar Assiut University, Assiut, Egypt

**Keywords:** Laparoscopic cholecystectomy, Indocyanine green, Intravenous injection, Intrabiliary injection, Liver fluorescence, Operative time

## Abstract

**Background:**

Laparoscopic cholecystectomy is associated with the risk of iatrogenic biliary structure injury. Therefore, indocyanine green fluorescence dye is used to provide better visualization of the different biliary structures to avoid this type of injury. However, there is still a debate regarding the best route of ICG administration—either intrabiliary or intravenous. This meta-analysis aims to compare the two techniques in terms of biliary structure visualization, bile duct injury rate, and operative time.

**Method:**

We conducted the meta-analysis following the PRISMA 2020 checklist, and the protocol was prospectively registered in PROSPERO (CRD420251078787) on 22 June 2025. We systematically searched PubMed, Scopus, Web of Science, and the Cochrane Library for clinical studies comparing the two techniques of dye injection. We included English-language clinical studies of any design (randomized controlled trials, prospective, retrospective, and case-control designs) published up to May 2025 that met our predefined eligibility criteria. Screening of eligible studies was conducted using Ryyan software. Data were extracted and analyzed using RevMan software version 5.4.1. We performed subgroup analyses based on study design. We assessed the quality of included studies using the risk of bias 2 tool (ROB 2) and the Newcastle-Ottawa scale (NOS). The certainty of evidence for all primary outcomes was appraised using the GRADE approach.

**Result:**

Four studies with a sample size of 178 patients were included: two retrospective studies, one randomized controlled trial, and one case-control study. Of these, 86 patients (48.3%) received intrabiliary ICG injection and 92 (51.7%) received intravenous injection. No significant differences were found between the two groups regarding biliary anatomy visualization before or after dissection of Calot’s triangle, including cystic duct (CD), common bile duct (CBD), and common hepatic duct (CHD) visualization (all *p* > 0.05). Liver fluorescence was significantly higher in the intravenous group (RR = 0.08, 95% CI 0.03–0.21, *p* < 0.00001). Operative time was significantly longer in the intrabiliary group (MD = 8.80 min, 95% CI 1.05–16.56, *p* = 0.03). No cases of bile duct injury were reported in either group. According to GRADE, the certainty of evidence across all primary outcomes was rated as very low.

**Conclusion:**

This meta-analysis explored the hypothesis that the route of indocyanine green (ICG) injection may influence biliary visualization during laparoscopic cholecystectomy. Intrabiliary and intravenous injections provided comparable visualization, with differences observed in liver fluorescence and operative time; lower liver fluorescence with intrabiliary injection may enhance visual clarity, while operative time was slightly longer. The outcome “bile duct injury” remains non-informative, as no events occurred in either group, precluding any comparative inference. Given the very low certainty of evidence due to study limitations, small sample sizes, few events, heterogeneity, and imprecision, these findings are hypothesis-generating only, and larger, well-designed randomized trials are needed to confirm their clinical relevance.

**Supplementary Information:**

The online version contains supplementary material available at 10.1186/s12893-026-03529-4.

## Background

Gallbladder disease is commonly associated with concomitant gallstones. It is a prevalent disease that needs surgical management [1]. Despite laparoscopic cholecystectomy (LC) having become the cornerstone of management due to its benefits compared to open surgical intervention, it carries a danger of major complications, especially iatrogenic biliary tract injury [2]. Bile duct injury is a potentially critical complication leading to marked morbidity and requiring further procedures [3].

To avoid these types of complications, intraoperative cholangiography (IOC) has been used during LC to give better visualization of biliary anatomy [4]. IOC is conducted by accessing the cystic duct and injecting an adequate contrast substance required for X-ray imaging [5]. However, IOC has its drawbacks, including prolonged operative time, technical difficulties, and risk of exposure to radiation and contrast material [6]. These drawbacks prompted the development and usage of alternative imaging methods. Intraoperative indocyanine green (ICG) fluorescence imaging has emerged as a promising alternative to IOC [7]. ICG is a fluorescent dye that, when injected intravenously, is released into the bile and fluoresces under near-infrared light, allowing for real-time delineation of biliary structures without exposure to radiation or contrast substances [8]. This method is regarded as non-invasive and may potentially improve surgical safety and reduce operation time [9]. Despite its benefits, it has limitations, for instance, its limited tissue penetration, which can present challenges in cases of obesity or severe inflammation [10].

Currently, there is no well-developed consensus on the best method of ICG injection for fluorescence cholangiography during LC [11]. Some studies have explored intravenous (IV) injection, which is the most common route [12]. Intrabiliary (IB) or intracholecystic (IC) injection has also been investigated [13]. Some evidence suggests that IB injection might provide more obvious images by reducing background hepatic fluorescence [14]. However, there is limited direct comparative evidence regarding the efficacy and outcomes between the two methods of injection [15].

This is the first meta-analysis comparing IV injection and IB injection of ICG dye during fluorescence cholangiography for patients undergoing LC regarding visualization of different biliary structures, operative time, and postoperative complications.

## Methods

We followed the Preferred Reporting Items for Systematic Reviews and Meta-Analyses (PRISMA) statement guidelines for conducting this systematic review and meta-analysis [16]. The protocol of this study was prospectively registered in PROSPERO (CRD420251078787) on 22 June 2025.

### Search strategy

PubMed, Scopus, Web of Science, and Cochrane Library were searched since inception up to May 2025 for relevant published clinical studies using the following keywords: indocyanine green, ICG, fluorescence cholangiography, near-infrared fluorescence, NIRF, fluorescent cholangiography, laparoscopic cholecystectomy, LC, intravenous injection, IV, systemic injection, intracholecystic injection, and intrabiliary injection. Additionally, we conducted manual screening of the reference lists of all included studies. The detailed search strategy is outlined in the supplementary Table 1.

### Selection criteria

All clinical studies, randomized controlled trials (RCTs), retrospective, prospective, and case-control studies published in English up to May 2025 that met the following criteria were included in our review:

1. Indication for laparoscopic cholecystectomy for any of the following causes: acute or chronic gallbladder disease, Single or multiple stones of the gallbladder, Gallbladder polyps, Gallbladder adenomyosis, gallbladder pancreatitis, and Biliary colics.
2. Including intrabiliary injection arm by any of the following methods of injection: direct gallbladder injection, percutaneous transhepatic gallbladder drainage (PTGBD) injection, and endoscopic nasobiliary drainage (ENBD) injection
3. Comparing the intrabiliary injection arm with the intravenous injection arm. Single-arm studies were excluded
4. Reporting at least one of the primary or secondary outcomes. The process of screening was conducted using Ryyan software by two independent reviewers. Conflict cases were resolved by discussion to reach a consensus [17].

### Data extraction

A data extraction Excel sheet was designed. After that, it was accessible to all authors. All authors participated in data extraction. Extracted data for each study included study ID (last name of first author and the publication year), country, study design, study groups, sample size, age, sex, BMI, detailed intervention, outcome measures, and key findings.

### Study outcomes

Our primary outcomes were as follows: cystic duct (CD) visualization before and after dissection of Calot’s triangle, common bile duct (CBD) visualization before and after dissection of Calot’s triangle, common hepatic duct (CHD) visualization before and after dissection of Calot’s triangle, and liver fluorescence. Our secondary outcomes were as follows: bile duct injury and operative time.

### Quality assessment

One reviewer performed a quality assessment, and the other reviewer revised the decision. Conflict cases were resolved by discussion to reach a consensus. We used the Cochrane risk of bias-2 (ROB 2) tool to assess the risk of bias in the included RCTs. Additionally, we used the Newcastle–Ottawa Scale (NOS) to assess the risk of bias in the included observational studies [18, 19].

### Certainty assessment

The certainty of evidence was assessed using the GRADE framework (Grading of Recommendations Assessment, Development and Evaluation). Two independent reviewers performed the ratings via GRADEpro Guideline Development Tool (McMaster University / Evidence Prime, 2025). Any disagreements were resolved through discussion. evidence was graded as “high”, “moderate”, “low”, or “very low” based on evaluations across five principal GRADE domains: risk of bias, inconsistency, indirectness, imprecision, and publication bias.

### Statistical analysis

Statistical analysis and all plots were conducted using RevMan software version 5.4.1. Weighted mean differences were used for continuous data, and risk ratios were used for dichotomous data, with 95% confidence intervals (CIs). A *p* < 0.05 was considered statistically significant, which was calculated by the *Z* test. Heterogeneity was evaluated using Cochran’s *Q* test, with significance at *p* < 0.1. The inconsistency across studies was quantified using the I² statistic. Significant heterogeneity was defined as *I²* > 50%. A random effect model was used in the analysis. subgroup analyses were performed based on study design to assess the robustness of the results across RCTs and observational studies.

## Results

### Study characteristics

**Fig. 1:**
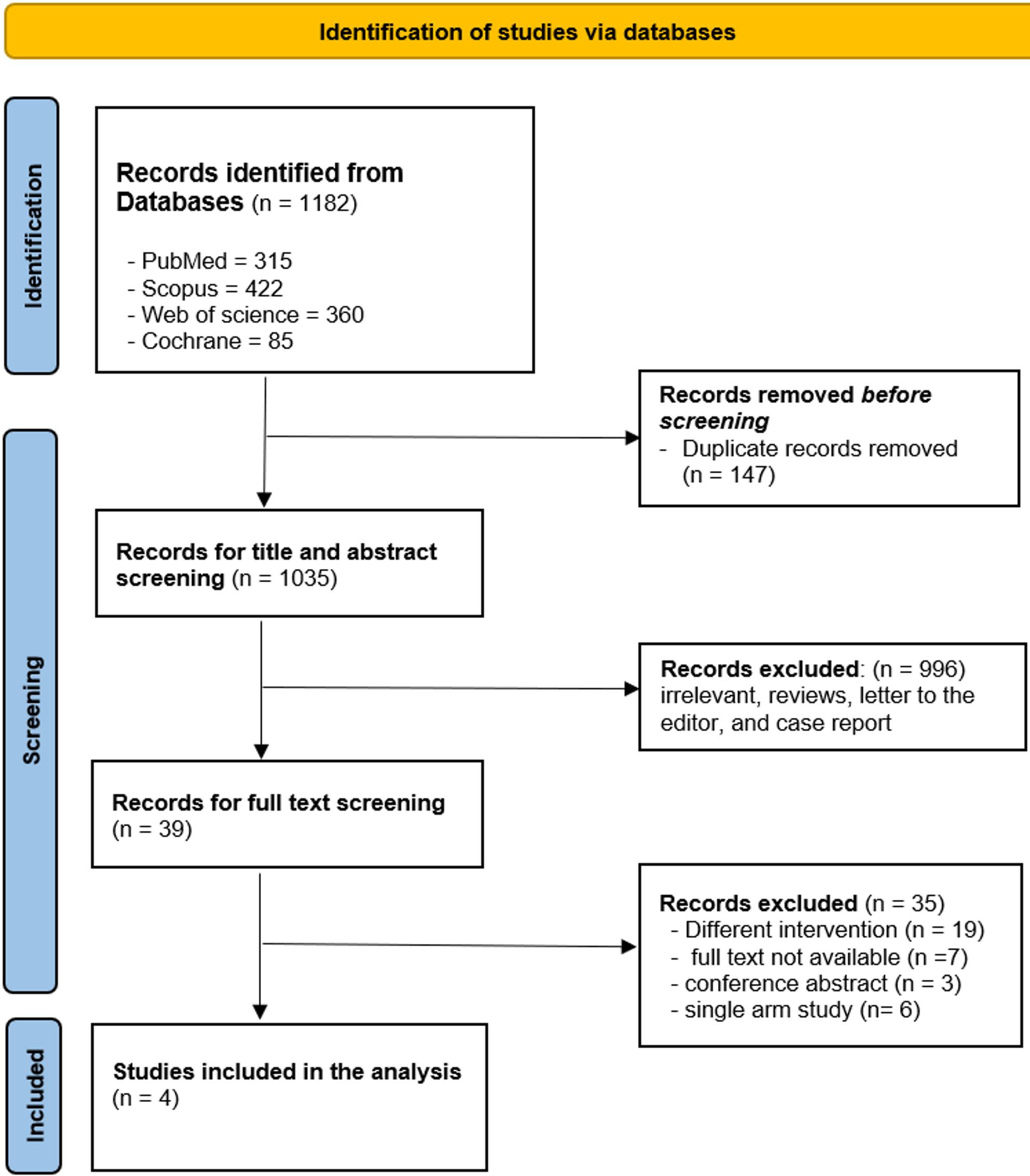
Prisma flow diagram

A total of four studies were included in the analysis [14, 20–22], of which 2 were retrospective studies, a single randomized controlled trial, and a single case-control study with a total sample size of 178 patients, of whom 86 (48.3%) underwent intrabiliary injection of ICG dye and 92 (51.7%) underwent intravenous injection. The detailed characteristics of the included studies are explained in Table 1. The screening process is explained in the flow diagram shown in Fig. 1.

**Table 1:** Study characteristics of included studies

Study	Year	Country	Study Design	Indications for LC	Groups	Sample size	Age (years) (Mean ± SD)	Sex Male: Female	BMI (Mean ± SD)	detailed injection (method / dose / time)
Elmeligy et al. [20]	2024	Egypt	RCT	Biliary colics Acute attack GB polyp GB pancreatitis	Intrabiliary	30	43.2 ± 12.9	6 to 24	28.48 ± 4.41	1.25 mg of ICG dissolved in 3 ml of saline, and the concentration was roughly 0.04 mg after each 1 ml was diluted by 9 ml of saline. To prevent dye leakage. The Veress needle was inserted through the abdominal wall and into the gall bladder fundus through the liver parenchyma (transhepatic), then the puncture site was cauterized.
					Intravenous	30	40.5 ± 11.5	8 to 22	31.92 ± 3.94	NR
Cai et al. [21]	2024	China	Retrospective cohort	Gallbladder polyps Single stone of the gallbladder Multiple stones in the gallbladder Gallbladder adenomyosis	Intrabiliary	27	47.04 ± 9.93	13 to 14	NR	3–5 mL of normal saline containing 10 mg of ICG was injected into the gallbladder lumen during surgery
					Intravenous	32	51.16 ± 12.52	18 to14	NR	10 mg of ICG was administered via peripheral intravenous injection 10 to 12 h before surgery
Castagneto-Gissey et al. [14]	2022	Italy	Case–Control Study	Symptomatic gallstone disease or acute/chronic cholecystitis	Intrabiliary	17	50 ± 19	6 to 11	24 ± 3	ICG (Verdye 25 mg/5 mL) was diluted in 10 mL of distilled, sterile water. The gallbladder was grasped at the level of its fundus and retracted cephalad, bringing it to the proximity of the anterior abdominal wall. At this point, a 27-gauge needle was used percutaneously to puncture the abdominal wall and the fundus of the gallbladder to inject ICG.
					Intravenous	18	55 ± 18	7 to 11	25 ± 3	ICG was administered intravenously approximately 45 min before surgery at a concentration of 0.01 mg/kg
Shibata et al. [22]	2021	Japan	Retrospective Observational Study	CBD stones or cholangitis One patient only underwent hepatectomy	Intrabiliary	12	62.8 for both groups	14 to 10 for both groups	NR	Injecting ICG directly into the bile duct is further divided into gallbladder (GB) puncture and bile duct injection. GB puncture involves puncturing the GB during surgery and injecting ICG (0.025 mg/mL). In contrast, with the bile duct injection method, ICG is injected into the bile duct via an extra biliary fistula tube inserted before surgery, such as that for PTGBD or ENBD.
					Intravenous	12				2.5 mg/body of ICG is administered 1 h before surgery

### Quality assessment and certainty of evidence

**Fig. 2:**
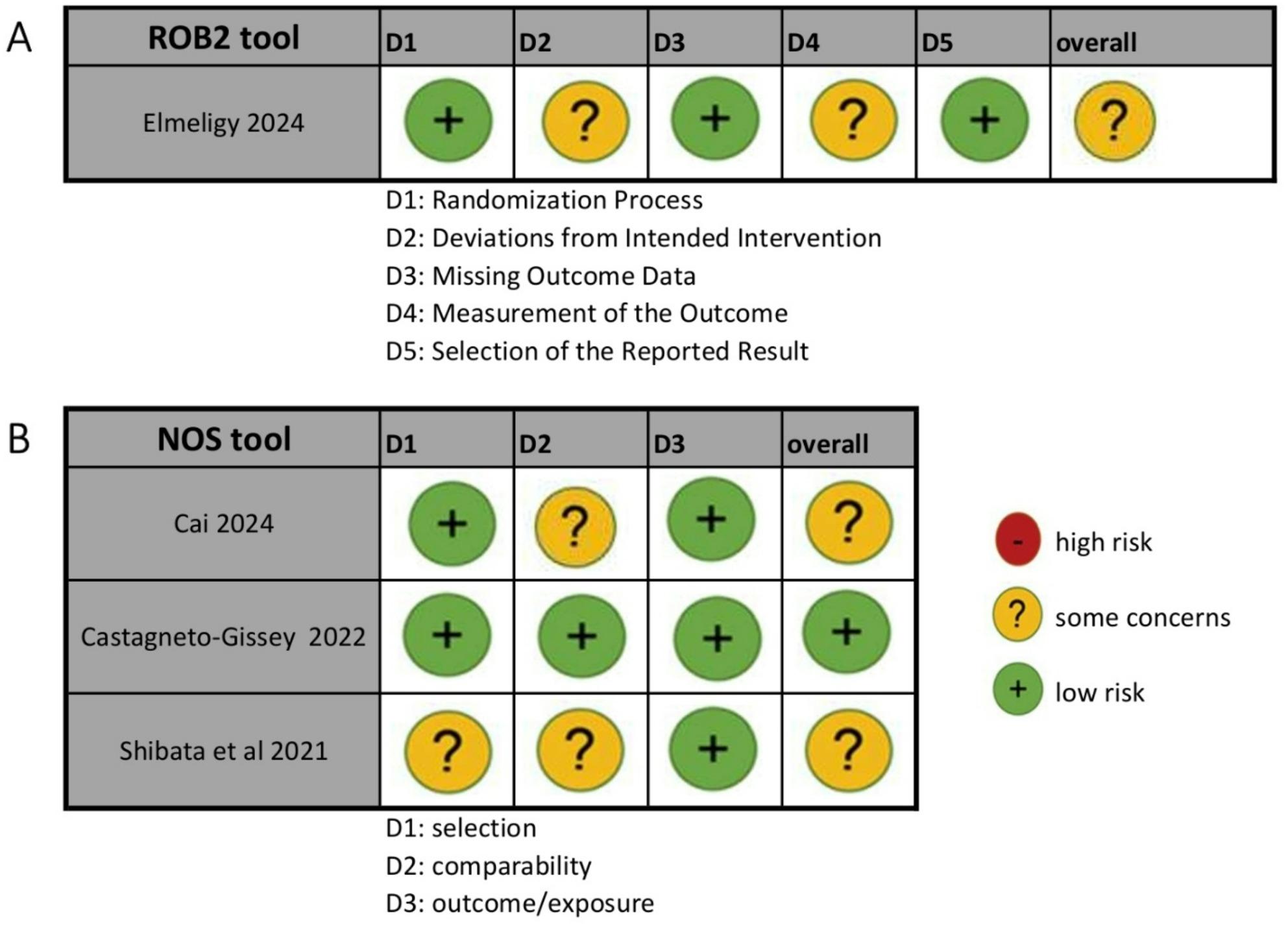
Quality assessment of risk of bias in the included studies: (**A**) RCTs assessed by RoB 2 and (**B**) observational studies assessed by NOS

The quality assessment was performed using the Cochrane risk of bias-2 (ROB 2) tool for randomized controlled trials and the Newcastle–Ottawa Scale (NOS) for observational studies. Among the four included studies, three demonstrated some concerns (Elmeligy 2024, Cai 2024, and Shibata 2021), while one study showed low risk of bias (Castagneto-Gissey 2022). Cai 2024 was rated with some concerns in the comparability domain due to a statistically significant baseline difference in preoperative diagnosis (*P* = 0.04) without multivariate adjustment. Shibata 2021 showed concerns in the selection domain because the cohort comprised patients with severe inflammation and anatomic variation, making it less representative of the typical LC population, and the study did not provide separate baseline characteristics for both groups. Elmeligy 2024 showed concerns in domains 2 and 4, as blinding of operating surgeons was not possible and blinding of outcome assessors was not reported (Fig. 2**).**

The overall certainty of evidence for the primary outcomes was then graded using the GRADE approach. All outcomes were rated as “very low” certainty. A comprehensive summary of these GRADE assessments is provided in Supplementary Table 2.

### Analysis

#### CD visualization before dissection of calot’s triangle

**Fig. 3:**
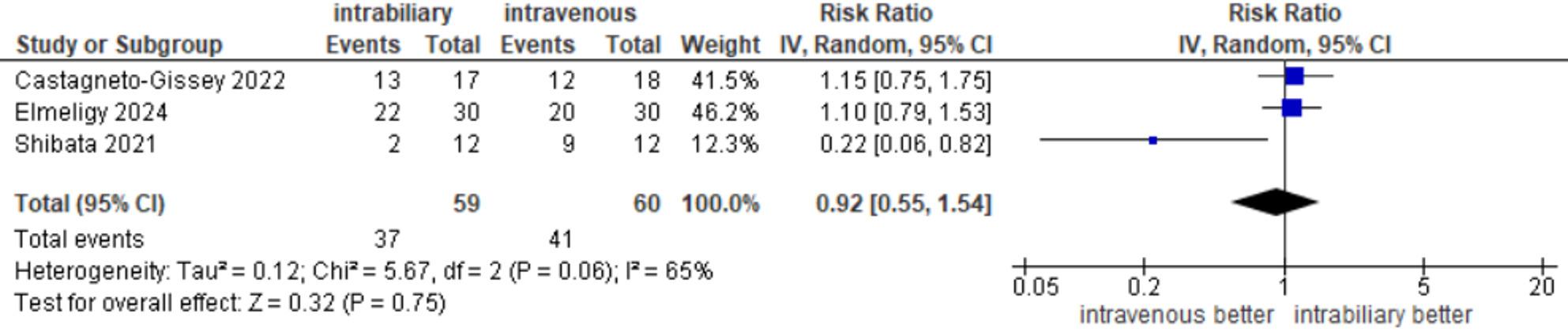
CD visualization before dissection of Calot's triangle

Outcomes of CD visualization before dissection were reported in three studies [14, 20, 22], with an incidence of 37 out of 59 (62.71%) in the intrabiliary group and 41 out of 60 (68.33%) in the intravenous group. The meta-analysis showed no significant difference between the two groups (RR = 0.95, 95% CI 0.68–1.32, *P* = 0.75), and there was significant moderate heterogeneity among the studies (I² = 65%, τ² = 0.12, *p* = 0.06) (Fig. 3).

#### CD visualization after dissection of calot’s triangle

**Fig. 4:**
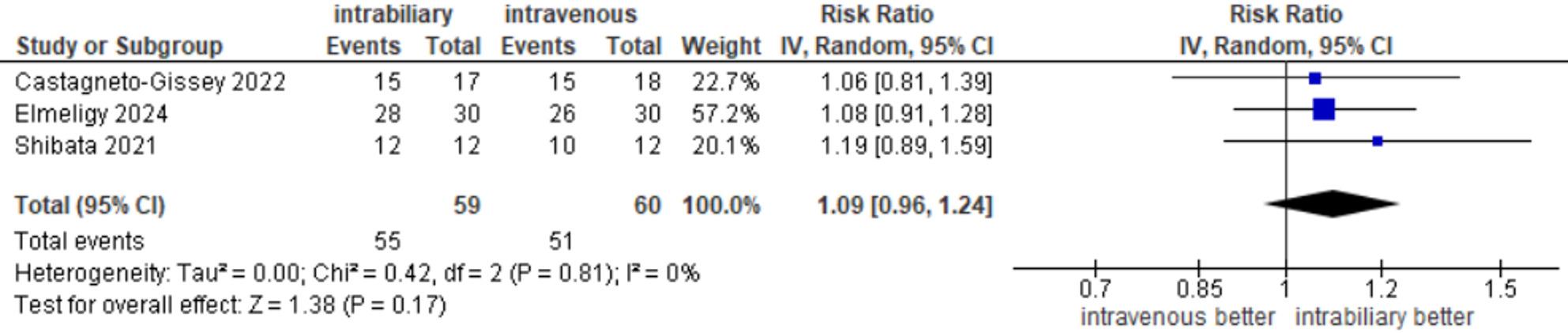
CD visualization after dissection of Calot's triangle

Outcomes of CD visualization before dissection were reported in three studies [14, 20, 22], with an incidence of 55 out of 59 (93.22%) in the intrabiliary group and 51 out of 60 (85%) in the intravenous group. The meta-analysis showed no significant difference between the two groups (RR = 1.09, 95% CI 0.96–1.24, *P* = 0.17), and there was no heterogeneity among the studies (I²=0%, τ²=0, *P* = 0.81) **(**Fig. 4**).**

#### CBD visualization before dissection of calot’s triangle

**Fig. 5:**
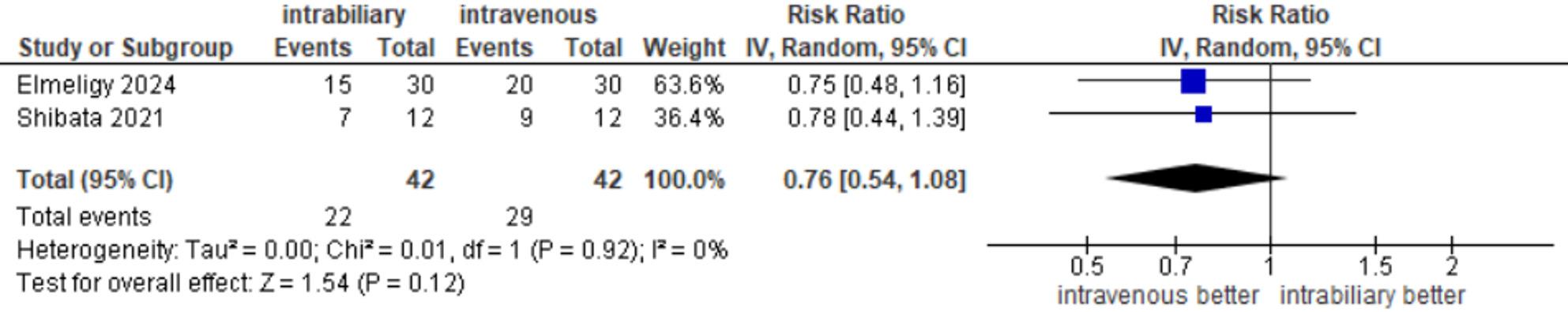
CBD visualization before dissection of Calot's triangle

Outcomes of the CBD visualization before dissection were reported in 2 studies [20, 22], with an incidence of 22 of 42 (52.38%) in the intrabiliary group and 29 of 42 (69.04%) in the intravenous group. The meta-analysis showed no significant difference between the two groups (RR = 0.76, 95% CI 0.54–1.08, *P* = 0.12), and there was no heterogeneity between the two studies (I²=0%, τ²=0, *p* = 0.92) **(**Fig. 5**)**.

#### CBD visualization after dissection of calot’s triangle

**Fig. 6:**
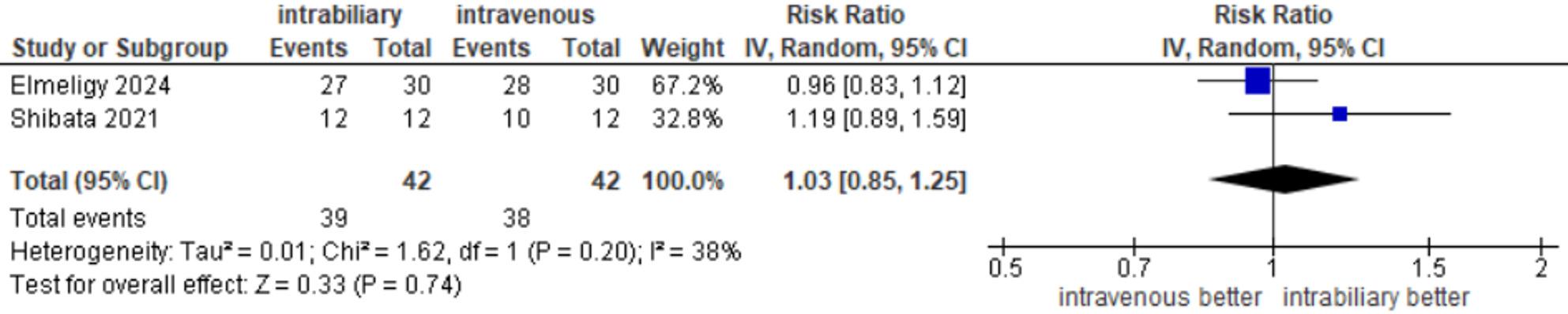
CBD visualization after dissection of Calot's triangle

Outcomes of the CBD visualization after dissection were reported in 2 studies [20, 22], with an incidence of 39 of 42 (92.85%) in the intrabiliary group and 38 of 42 (90.47%) in the intravenous group. The meta-analysis showed no significant difference between the two groups (RR = 1.03, 95% CI 0.85–1.25, *P* = 0.74), and there was an insignificant low heterogeneity between the two studies (I²=38%, τ²=0.01, *p* = 0.20) **(**Fig. 6**).**

#### CBD visualization

**Fig. 7:**
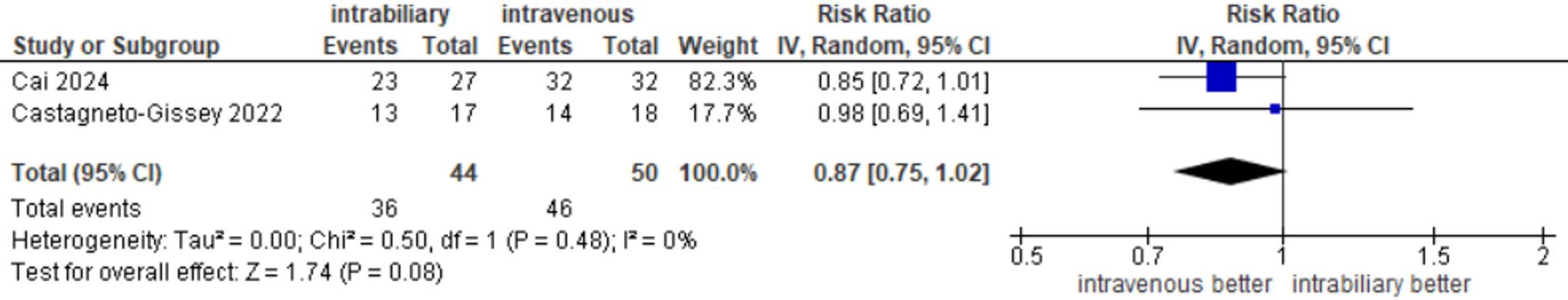
CBD visualization

Two of the included studies reported the outcome of CBD visualization without specifying whether these values were before or after the dissection of Calot’s triangle [14, 21]; therefore, they were analyzed separately. The incidence of visualization was 36 of 44 (81.81%) in the intrabiliary group and 46 of 50 (92%) in the intravenous group. The meta-analysis showed no significant difference between the two groups (RR = 0.87, 95% CI 0.75–1.02, *P* = 0.08), with no heterogeneity between the two studies (I² = 0%, τ² = 0, *p* = 0.48) **(**Fig. 7**).**

#### CHD visualization before dissection of calot’s triangle

**Fig. 8:**
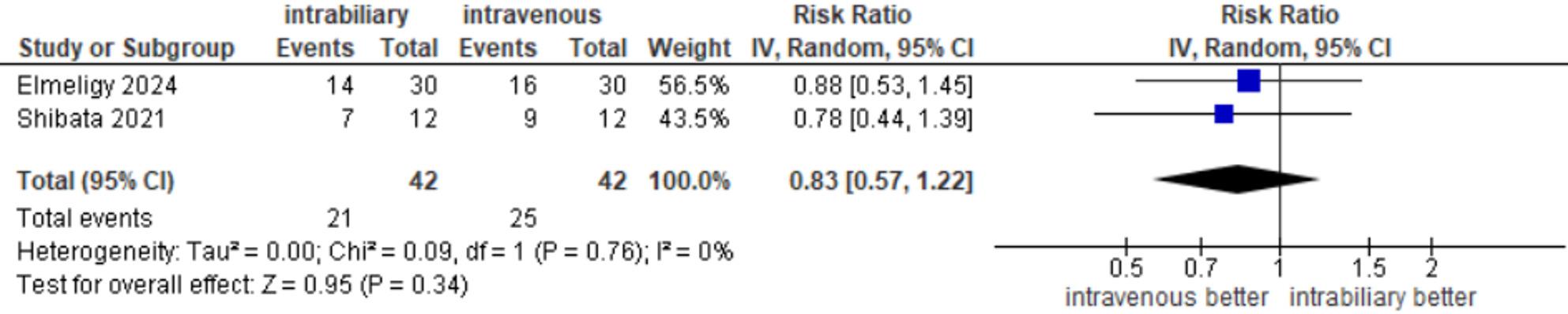
CHD visualization before dissection of Calot's triangle

Outcomes of the CHD visualization before dissection were reported in 2 studies [20, 22], with an incidence of 21 of 42 (50%) in the intrabiliary group and 25 of 42 (59.52%) in the intravenous group. The meta-analysis showed no significant difference between the two groups (RR = 0.83, 95% CI 0.57–1.22, *P* = 0.34), and there was no heterogeneity between the two studies (I²=0%, τ²=0, *P* = 0.76) **(**Fig. 8**).**

#### CHD visualization after dissection of calot’s triangle

**Fig. 9:**
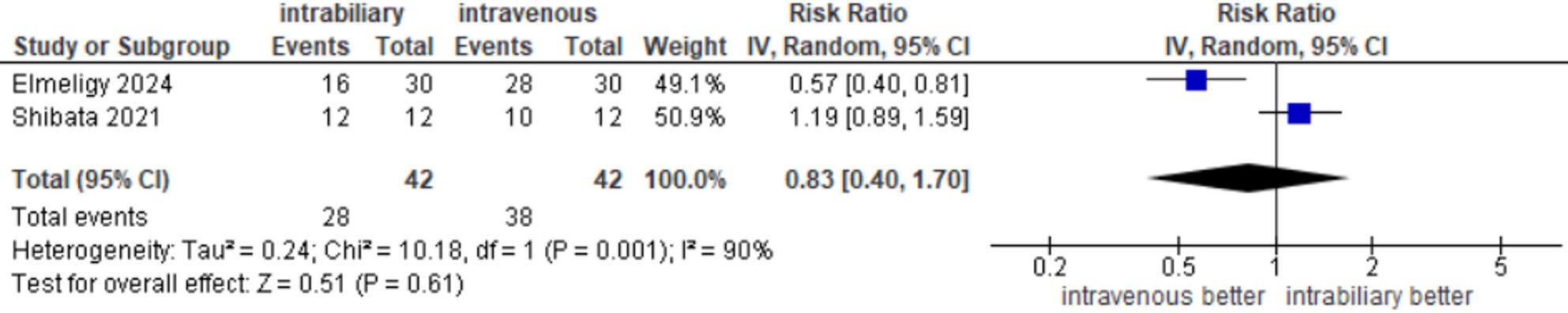
CHD visualization after dissection of Calot's triangle

Outcomes of the CHD visualization after dissection were reported in 2 studies [20, 22], with an incidence of 28 out of 42 (66.66%) in the intrabiliary group and 38 out of 42 (90.47%) in the intravenous group. The meta-analysis showed no significant difference between the two groups (RR = 0.83, 95% CI 0.40–1.70, *P* = 0.61), and there was significantly high heterogeneity between the two studies (I² = 90%, τ² = 0.24, *p* = 0.001) **(**Fig. 9**).**

#### Liver visualization (Fluorescence)

**Fig. 10:**
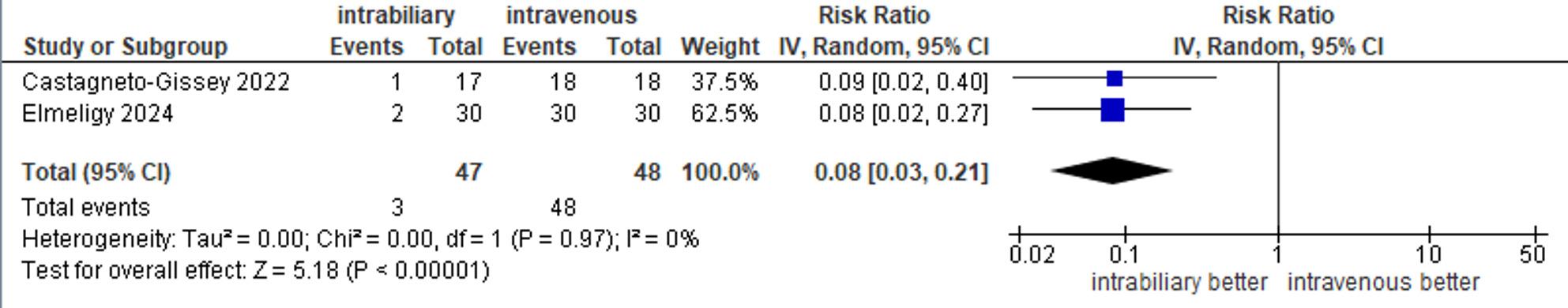
Liver Visualization (Fluorescence)

Outcomes of the liver visualization before dissection were reported in 2 studies [14, 20], with an incidence of 3 out of 47 (6.38%) in the intrabiliary group and 48 out of 48 (100%) in the intravenous group. these findings demonstrate that the intrabiliary approach is associated with substantially lower hepatic background fluorescence. In contrast, intravenous administration consistently results in greater liver visualization (RR = 0.08, 95% CI 0.03–0.21, *P* < 0.00001), with no heterogeneity between the two studies (I² = 0%, τ² = 0, *p* = 0.97) **(**Fig. 10**).**

#### Operative time

**Fig. 11:**
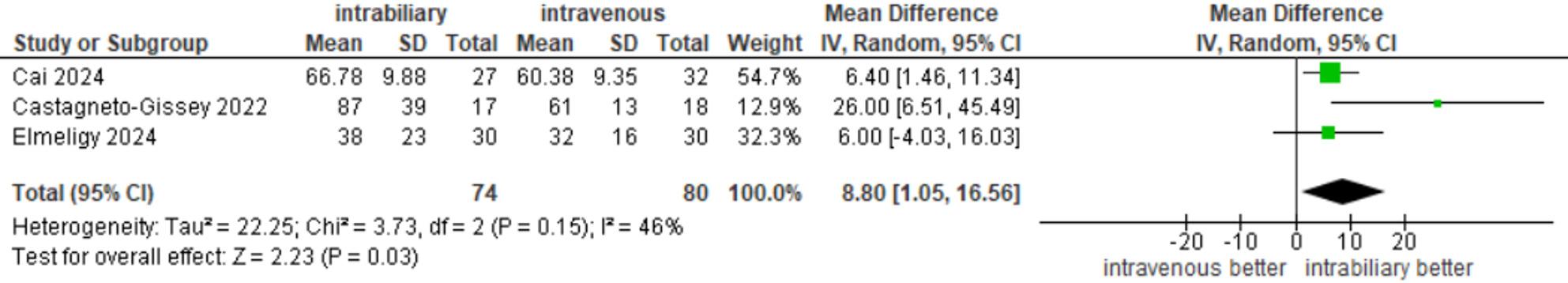
Liver Visualization (Fluorescence)

Outcomes of operative time were reported in 3 studies [14, 20, 21]. The meta-analysis showed significantly longer operative time in the intrabiliary injection group compared to the intravenous group (MD = 8.80, 95% CI 1.05–16.56, *P* = 0.03), and there was an insignificant low heterogeneity among the studies (I² = 46%, τ² = 22.25, *p* = 0.15) **(**Fig. 11**).**

#### 10 bile duct injury

Outcomes of bile duct injury were reported in 3 studies [14, 20, 21], with no events observed in either the intrabiliary group (0/74) or the intravenous group (0/80). Because both groups reported zero events, a comparative effect estimate cannot be calculated, and no conclusions can be drawn regarding differences between the interventions.

#### Subgrouping meta-analysis

We conducted Subgroup analysis based on study design (RCTS and observational studies) for CD visualization before dissection, CD visualization after dissection, and operative time.

Regarding subgroup analysis for CD visualization before dissection, both subgroups (RCTS and observational studies) demonstrated statistically insignificant differences in visualization of CD between the two methods of injection (RR = 1.10, 95% CI 0.79–1.53), (RR = 0.57, 95% CI 0.12–2.80, I² = 82%), respectively. The results of subgroup analysis were consistent with the overall estimate of both subgroups (RR = 0.92, 95% CI 0.55–1.54, *P* = 0.75) (Supplementary Fig. 1A).

Regarding subgroup analysis for CD visualization after dissection, both subgroups (RCTs and observational studies) demonstrated a statistically insignificant difference in visualization of CD between the two methods of injection (RR = 1.08, 95% CI 0.91–1.28) and (RR = 1.12, 95% CI 0.92–1.36), respectively. The results of the subgroup analysis were consistent with the overall estimate (RR = 1.09, 95% CI 0.96–1.25, *P* = 0.17) for both subgroups (Supplementary Fig. 1B).

Regarding subgroup analysis for operative time, both subgroups (RCTS and observational studies) demonstrated statistically insignificant difference in operative time between the two methods of injection (MD = 6.00 min, 95% CI -4.03–16.03), (MD = 13.84, 95% CI -4.80–32.48, I² = 73%) respectively. However, the pooled estimate of both subgroups demonstrated a statistically significant increase in operative time in the intrabiliary group compared to the intravenous group (MD = 8.80 min, 95% CI 1.05–16.56, I² = 46%) (Supplementary Fig. 1C).

The two included studies in CBD visualization before dissection of Calot’s triangle, CBD visualization not determined before or after dissection, and CHD visualization before dissection of Calot’s triangle showed risk ratios below 1, indicating a direction favoring intravenous injection; however, confidence intervals crossed the line of no effect. Therefore, the pooled estimate showed no statistically significant difference between intrabiliary and intravenous injection **(**Figs. 5 and 7**).**

Regarding CBD visualization after dissection of Calot’s triangle and CHD visualization after dissection of Calot’s triangle, Elmeligy 2024 showed risk ratios below 1, favoring intravenous injection. Shibata 2021 reported risk ratios above 1, favoring intrabiliary injection. However, their confidence intervals crossed the line of no effect, demonstrating an insignificant difference between the two interventions **(**Fig. 6**).**

Regarding liver visualization (FLOURESENCE), Elmeligy 2024 and Shibata 2021 showed risk ratios below 1, with confidence intervals that didn’t cross the line of no effect, demonstrating the statistically significant benefit of intravenous injection over intrabiliary injection **(**Fig. 10**)**.

## Discussion

Gallbladder disease, presenting commonly as gallstones and inflammation (cholelithiasis and cholecystitis), represents a marked global health burden and is a leading cause of hospital admissions related to gastrointestinal diseases [23]. Laparoscopic cholecystectomy (LC) is known to be the gold standard and one of the most frequently performed abdominal surgeries for symptomatic gallbladder disease worldwide [24]. Although laparoscopic cholecystectomy (LC) has several benefits, it is associated with a considerable risk of iatrogenic bile duct injuries (BDI), with reported incidences ranging from 0.08% to 1.5% [25]. These injuries are a major issue due to their association with notable morbidity and mortality, decreased quality of life, and substantial healthcare costs, including complications such as biliary stricture, sepsis, and recurrent cholangitis [25]. In response to these challenges, near-infrared cholangiography (NIRC) with indocyanine green (ICG) has emerged as a promising solution to enhance intraoperative visualization of the biliary tree, achieve a critical view of safety, and reduce complications like biliary injuries [26].

Liu et al. [27] is an animal study that was implemented on seven swine. This study found that near-infrared fluorescence cholecysto-cholangiography using a direct intragallbladder injection of ICG dye provides more real-time guidance to identify biliary structures in comparison with intravenous injection of the dye. Compared to systemic injection, this method offers a faster and better contrasted enhancement of Calot’s triangle and the gallbladder itself.

A limited number of human studies have compared the two injection techniques, and their results have also been conflicting. Therefore, this meta-analysis aims to compare the two injection techniques in terms of different biliary tree structure visualization, operative time, and postoperative complications.

This meta-analysis demonstrated no statistically significant differences between the two injection methods for cystic duct visualization, common bile duct visualization, and common hepatic duct visualization. However, statistically significant differences were observed for liver visualization, favoring the intravenous group compared with the intrabiliary group (RR = 0.08, 95% CI 0.03–0.21, *P* < 0.00001), and for operative time, which was longer in the intrabiliary injection group compared with the intravenous group (MD = 8.80, 95% CI 1.05–16.56, *P* = 0.03). Bile duct injury was reported in three studies, with zero events in both groups, precluding calculation of a comparative effect.Given the small sample size, few outcome events, and substantial clinical and methodological heterogeneity across the included studies, these findings should be interpreted with caution and considered hypothesis-generating only. Furthermore, the GRADE assessment rated the certainty of evidence for all primary outcomes as very low due to study limitations, inconsistency, and imprecision; therefore, no conclusions regarding clinical superiority or practice-changing implications can be drawn.The reason for the increased liver fluorescence in the intravenous injection group is that intravenous ICG injection is delivered systemically, allowing hepatic uptake and parenchymal accumulation of the dye before biliary excretion. In contrast, intrabiliary injection introduces the dye directly into the biliary system, bypassing the hepatic parenchyma, and therefore yields minimal liver fluorescence. This increase in liver fluorescence has both advantages and disadvantages. One of the advantages is that it indicates the dye has reached the normal biliary ducts, enhancing the visualization of the biliary anatomy and helping to reduce the risk of biliary tree injury. On the other hand, one of the disadvantages is that excessive liver fluorescence may create background noise, making it more difficult to identify smaller ducts, particularly in cases with inflammation or adhesions [14, 20].

The increase in operative time with intrabiliary injection is mainly due to the additional technical steps required during surgery. These include accessing and puncturing the gallbladder or cystic duct to inject the dye, confirming correct catheter or needle placement, and managing potential dye leakage, which may require extra suction or irrigation. Intrabiliary injection also demands more preparation and carries a higher risk of technical difficulties compared to intravenous injection [14].

In the subgroup analyses, neither study design group showed a meaningful difference in CD visualization before or after dissection between the two injection methods, and findings were consistent with the overall effect. For operative time, although each subgroup alone showed no significant difference, the pooled analysis indicated a slight increase in operative time with intrabiliary injection.

CBD visualization before dissection of Calot’s triangle, CBD visualization not determined before or after dissection, and CHD visualization before dissection of Calot’s triangle demonstrated risk ratios below 1, favoring intravenous injection; although the confidence intervals crossed the line of no effect, resulting in no significant pooled difference.

For CBD and CHD visualization after dissection, the two studies showed opposite directions of effect; however, both were non-significant. In contrast, liver fluorescence consistently favored intravenous injection, with both studies showing significant risk ratios below 1 and confidence intervals that did not cross the line of no effect. Nevertheless, the robustness of these subgroup findings is limited by the very low certainty of evidence as per GRADE, restricting the confidence with which these differences can be generalized to broader clinical practice.

One of the strengths of this meta-analysis is that it includes all types of study designs—covering randomized controlled trials, retrospective studies, and case-control studies—with a total of four studies involving a sample size of 178 patients, aiming to minimize bias through comprehensive study inclusion.

However, the limitations of this meta-analysis must be acknowledged, including the inherent biases associated with the inclusion of retrospective studies, potential heterogeneity among the included studies. Second, the limited number of included studies with small sample sizes makes the heterogeneity statistics and subgroup effects unstable when the number of studies is small [1, 2]. Additionally, we did not include grey literature such as protocols from clinical trial registries or sources indexed through Google Scholar. Furthermore, although our protocol restricted inclusion to English-language studies, this criterion did not ultimately impact study selection, as no eligible non-English articles were identified during screening. Collectively, these limitations contributed to the very low GRADE certainty ratings, emphasizing the need for larger, high-quality trials to increase confidence in the observed effects.

In conclusion, this systematic review and meta-analysis was conducted to explore the hypothesis that the route of indocyanine green (ICG) injection may influence biliary anatomy visualization during laparoscopic cholecystectomy. The findings indicate that intrabiliary and intravenous ICG injection techniques provide comparable visualization of the biliary anatomy. Differences were observed in liver fluorescence characteristics and operative time between the two injection routes. Notably, lower liver fluorescence associated with intrabiliary injection may reduce background hepatic signal, potentially improving visual clarity of the biliary structures and facilitating more precise anatomical identification during surgery. Additionally, operative time was observed to be slightly longer with intrabiliary injection. The outcome “bile duct injury” remains non-informative, as no events occurred in either group, precluding any comparative inference. These observations should be interpreted with caution and do not imply clinical superiority.

According to the GRADE assessment, the certainty of evidence for all primary outcomes was rated as very low due to study limitations, inconsistency, imprecision, small sample sizes, few outcome events, and heterogeneity across study designs. Therefore, the present findings should be considered hypothesis-generating only. Larger, well-designed, adequately powered randomized controlled trials are required to determine whether these observed differences translate into clinically meaningful benefits.

## Supplementary Information

Supplementary Material 1: Fig 1. Subgroup analysis. Table 1. Details of the search strategy. Table 2: GRADE assessment table for certainty of evidence.

## Data Availability

All data supporting the findings of this review are included in the article and its supplementary materials. Additional datasets—including screening logs and data extraction sheets—are available from the corresponding author upon reasonable request.

## Abbreviations

BDI: Bile Duct Injury
BMI: Body Mass Index
CBD: Common Bile Duct
CD: Cystic Duct
CHD: Common Hepatic Duct
ENBD: Endoscopic Nasobiliary Drainage
IB: Intrabiliary
ICG: Indocyanine Green
IOC: Intraoperative Cholangiography
IV: Intravenous
LC: Laparoscopic Cholecystectomy
MD: Mean Difference
NIRF: Near-Infrared Fluorescence
NOS: Newcastle–Ottawa Scale
PTGBD: Percutaneous Transhepatic Gallbladder Drainage
RCT: Randomized Controlled Trial
ROB 2: Risk of Bias 2 Tool
RR: Risk Ratio
