# Supplementary material for: Intrabiliary injection of indocyanine green dye (ICG) versus intravenous injection for patients undergoing laparoscopic cholecystectomy for symptomatic gallbladder diseases: A systematic review and meta-analysis
Source: BMC Surg. 2026 Feb 16;26:160. doi: 10.1186/s12893-026-03529-4 (PMC12933992; doi:10.1186/s12893-026-03529-4)
Supplement: Supplementary file 1 — Supplementary Material 1: Fig 1. Subgroup analysis. Table 1. Details of the search strategy. Table 2: GRADE assessment table for certainty of evidence. [file 12893_2026_3529_MOESM1_ESM.docx]

***Supplementary Material***

***Title:*** **Intrabiliary injection of indocyanine green dye (ICG) versus intravenous injection for patients undergoing laparoscopic cholecystectomy for symptomatic gallbladder diseases: Systematic Review and Meta-Analysis**

Mohamed Gamal^1^, Sohieb Hedawy^2^*

| **Supplementary Content:** | | |
| --- | --- | --- |
| **a. Tables:** | | **Page** |
| **1** | **Supplementary Table 1.** Details of the search strategy | **2** |
| **2** | **Supplementary Table 2.** GRADE Summary of findings table | **3** |
| **b. Figure:** | | **Page** |
| **1** | **Supplementary Fig. 1** Subgroup analysis for**: (A)** CD visualization before dissection of Calot's triangle**, (B)** CD visualization after dissection of Calot's triangle**,** and **(C)** operative time. | **3** |

**Supplementary Table 1.** Details of the search strategy

| **Databases** | **Restrictions** | **Term** | **Number of items found** |
| --- | --- | --- | --- |
| PubMed | Title and abstract | ("Laparoscopic Cholecystectomy" OR "Celioscopic Cholecystectomy" OR "LC")  AND  ("Indocyanine Green" OR "ICG" OR "ICG dye" OR "Indocyanine green dye" OR "ICG fluorescence" OR "Near-infrared fluorescent dye" OR "Indocyanine green dye" OR "ICG fluorophore" OR "NIR dye”) | 315 |
| Scopus | Title, abstract, and keyword |  | 422 |
| WOS | Topic |  | 360 |
| Cochrane | Title, abstract, and keywords |  | 85 |
| Total | |  | 1182 |

**PubMed**: Public/Publisher MEDLINE database; **WOS**: Web of Science; **Cochrane**: Cochrane Central Register of Controlled Trials; **Scopus**: Elsevier's abstract and citation database.

| **Supplementary Table 2.** GRADE Summary of findings table | | | | | |  |
| --- | --- | --- | --- | --- | --- | --- |
| Outcomes | **Anticipated absolute effects^*^** (95% CI) | | Relative effect (95% CI) | № of participants (studies) | Certainty of the evidence (GRADE) | |
|  | **Risk with Intravenous** | **Risk with Intrabiliary** |  |  |  |  |
| CD visualization before dissection | 683 per 1,000 | **629 per 1,000** (1,000 to 376) | **RR 0.92** (0.55 to 1.54) | 119 (3 non-randomised studies) | ⨁◯◯◯ Very low^a,b,c,d^ | |
| CD visualization after dissection | 850 per 1,000 | **927 per 1,000** (1,000 to 816) | **RR 1.09** (0.96 to 1.24) | 119 (3 non-randomised studies) | ⨁◯◯◯ Very low^a,c,d^ | |
| CBD visualization before dissection | 690 per 1,000 | **525 per 1,000** (746 to 373) | **RR 0.76** (0.54 to 1.08) | 84 (2 non-randomised studies) | ⨁◯◯◯ Very low^a,c,d^ | |
| CBD visualization after dissection | 905 per 1,000 | **932 per 1,000** (1,000 to 769) | **RR 1.03** (0.85 to 1.25) | 84 (2 non-randomised studies) | ⨁◯◯◯ Very low^a,c,d^ | |
| CHD visualization before dissection | 595 per 1,000 | **494 per 1,000** (726 to 339) | **RR 0.83** (0.57 to 1.22) | 84 (2 non-randomised studies) | ⨁◯◯◯ Very low^a,c,d^ | |
| CHD visualization after dissection | 905 per 1,000 | **751 per 1,000** (1,000 to 362) | **RR 0.83** (0.40 to 1.70) | 84 (2 non-randomised studies) | ⨁◯◯◯ Very low^a,b,c,d^ | |
| Liver visualization ( FLOURESENCE ) | 1,000 per 1,000 | **80 per 1,000** (210 to 30) | **RR 0.08** (0.03 to 0.21) | 95 (2 non-randomised studies) | ⨁◯◯◯ Very low^a,c^ | |

a. Some studies have some concerns risk of bias; b. There is significant heterogeneity; c. small sample size; d. The confidence interval contains benefit and harm.


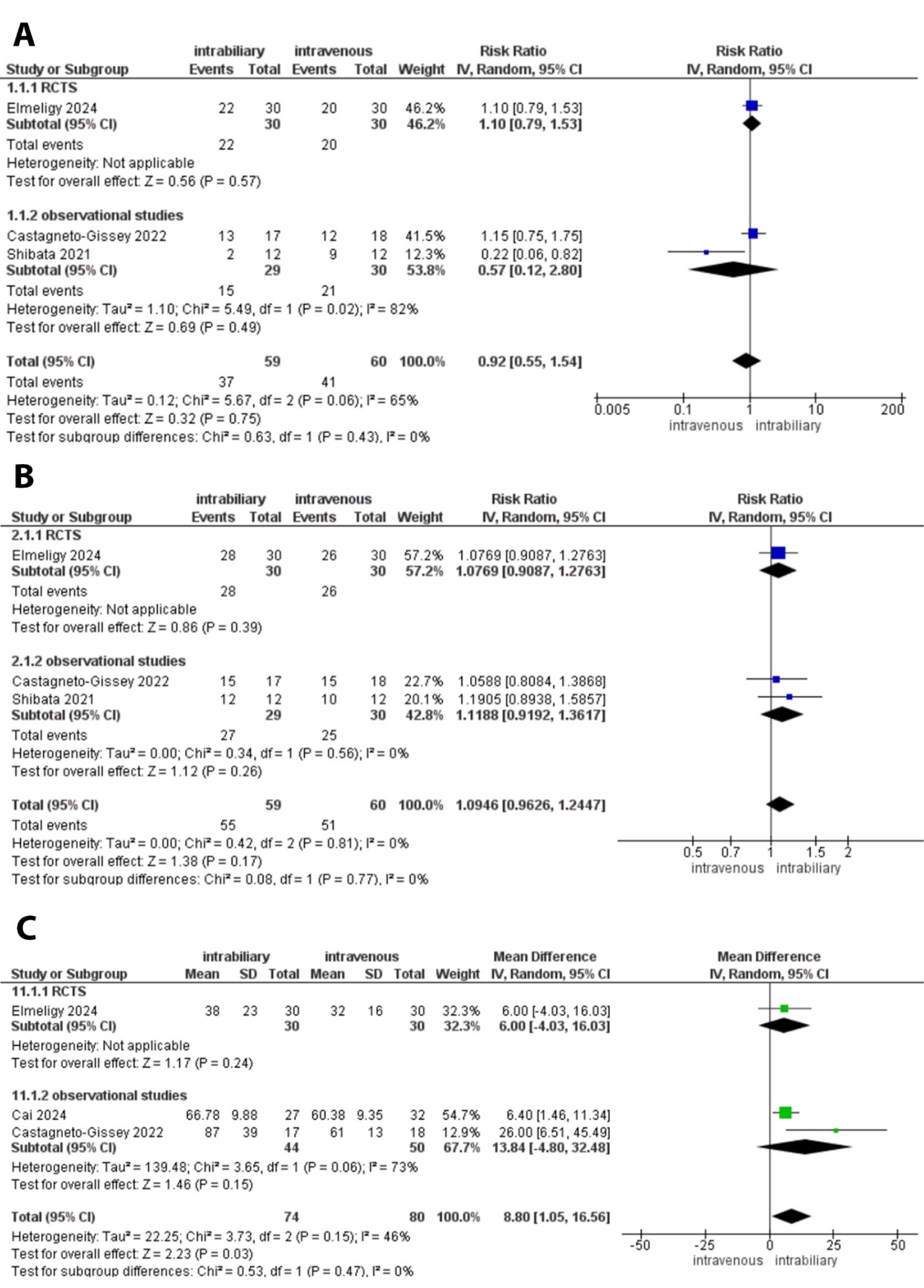


**Supplementary Fig 1.** Subgroup analysis for**: (A)** CD visualization before dissection of Calot's triangle**, (B)** CD visualization after dissection of Calot's triangle**,** and **(C)** operative time
